# Relationship Between Patient-Reported Outcome Measures and the Severity of Chronic Obstructive Pulmonary Disease in the Context of an Innovative Digitally Supported 24-Hour Service: Longitudinal Study

**DOI:** 10.2196/10924

**Published:** 2019-06-02

**Authors:** Signe Lindskrog, Karl Bang Christensen, Richard H Osborne, Søren Vingtoft, Klaus Phanareth, Lars Kayser

**Affiliations:** 1 Department of Public Health University of Copenhagen Copenhagen Denmark; 2 Faculty of Health, Arts and Design Swinburne University of Technology Hawthorn Australia; 3 Region Zeeland Sorø Denmark

**Keywords:** health literacy, empowerment, patient reported outcome measures, self-reported mental and physical health, health literacy questionnaire, health education impact questionnaire, SF-36, epital living lab, chronic obstructive pulmonary disease

## Abstract

**Background:**

Individuals with chronic obstructive pulmonary disease (COPD) live with the burden of a progressive life-threatening condition that is often accompanied by anxiety and depression. The severity of the condition is usually considered from a clinical perspective and characterized according to the Global Initiative for Chronic Obstructive Lung Disease (GOLD) classification of severity (1-4) and a risk assessment (A through D) that focuses on the patient’s symptoms and number of exacerbations, but information about perceived health or ability to manage the condition are rarely included.

**Objective:**

We evaluated 3 patient-reported outcome measurements (PROMs) to examine how these can be used to report on individuals with COPD who were supported by a digitally assisted intervention that aims to increase the patient’s management of their condition to improve their well-being.

**Methods:**

A total of 93 individuals with COPD were enrolled. At baseline and after 6 and 12 months, we measured self-reported self-management (Health Education Impact Questionnaire, heiQ) and health literacy (Health Literacy Questionnaire, HLQ), and physical and mental health (Short Form-36, SF-36) PROMs were collected. The scores of the 19 PROM dimensions were related to COPD severity, that is, GOLD risk assessment, pulmonary function at entry, and number of exacerbations of a period up to 12 months. The initial PROM scores were also compared with pulmonary function, exacerbations, and GOLD risk assessment to predict the number of contacts within the first 90 days.

**Results:**

At baseline, 2 dimensions from heiQ and SF-36 Physical health differed significantly between GOLD risk factor groups, indicating more distress and poorer attitudes and health status with increasing severity (GOLD risk assessment). Pulmonary function (FEV1) was negatively associated with the severity of the condition. After 6 months, we observed an increase in heiQ6 (skill and technique acquisition) and a reduction in emotional distress. The latter effect persisted after 12 months, where heiQ4 (self-monitoring and insight) also increased. HLQ3 (actively managing my health) decreased after 6 and 12 months. The number of exacerbations and the GOLD risk factor assessment predicted the number of contacts during the first 90 days. Furthermore, 2 of the PROMS heiQ6 (skill and technique acquisition) and HLQ8 (ability to find good health information) evaluated at baseline were associated with the number of contacts within the first 90 after enrollment. The pulmonary function was not associated with the number of contacts.

**Conclusions:**

Our data suggest that selected dimensions from HLQ, heiQ, and SF-36 can be used as PROMs in relation to COPD to provide researchers and clinicians with greater insight into how this condition affects individuals’ ability to understand and manage their condition and perception of their physical and mental health. The PROMs add to the information obtained with the clinical characteristics including the GOLD risk factor assessment.

**International Registered Report Identifier (IRRID):**

RR2-10.2196/resprot.6506

## Introduction

### Background

People diagnosed with chronic obstructive pulmonary disease (COPD) are affected by the burden of living with a deteriorating life-threatening condition. This condition is characterized by breathlessness, with a feeling of a burden that those living with COPD need to learn how to coexist with and have control over [1]. Increasing severity of COPD may result in a decreased level of activity, experience of isolation, increasing dependency on health professionals, and development of distress and often result in comorbidities such as anxiety and depression [2]. The severity of the condition is often described according to the Global Initiative for Chronic Obstructive Lung Disease (GOLD) classification of severity, which builds on clinical characteristics and numbers of exacerbations, but no information about how the condition affects the patient’s well-being, ability to manage their condition, or need of support is currently evaluated [3]. Here, the usage of psychometric instruments used as patient-reported outcome measures (PROM) may fill in a gap and contribute to a better understanding of how it is to live with COPD.

We here report on the utility of 3 validated PROMs to better understand people living with COPD and how a proactive and person-centered intervention, the Epital living lab, may influence the participants’ ability to manage their condition and well-being.

The Epital living lab was established in 2013 to develop and test a new way to offer services to people living with COPD for increasing their independence and well-being and relieving the burden of the condition and treatment. The findings of the Epital living lab is reported elsewhere [4]. The Epital living lab was organized to provide services in a proactive way, taking full advantage of digitalization. The participants are actively involved in the management of their own condition and are supported by a 24/7 response and coordination center, which connects participants to all services they need and also initiate treatments in collaboration between the participants and care providers including medical doctors. This creates an environment where the participants are not restricted by or confined to predefined schedules of monitoring but can use the provided technologies to monitor themselves and consult health care professionals whenever they wish to.

It was hypothesized that this redesign with its involvement of the participants in taking care of their own condition would increase their health literacy, ability to manage their condition, and ultimately their physical and mental well-being.

### Patient-Reported Outcome Measures

To evaluate how living with COPD affects the participants as well as the impact of the Epital living lab intervention, we used a set of multidimensional PROMs that assess diverse patient-centered outcomes—the Health Literacy Questionnaire (HLQ), Health Education Impact Questionnaire (heiQ), and the Short Form-36 (SF-36) [5-7].

The HLQ covers 9 conceptually distinct dimensions of health literacy. These dimensions reflect important elements from the perspective of the general population, health care providers, as well as policy makers. HLQ was developed using a validity-driven approach [7] to be a sensitive measure for the evaluation of interventions [7]. Recently, 2 of the 9 dimensions, HLQ6 (ability to actively engage with health care providers) and HLQ9 (ability to understand health information), were applied in 29,473 people within the Danish National Health Survey [8]. It has been validity tested in the Danish language [9] and other European languages and was found to have robust psychometric properties [10,11].

The heiQ is a widely used PROM to evaluate patient education interventions and self-management among people with a broad range of chronic conditions including COPD [12,13]. It was also developed using a validity-driven approach [6] and measures proximal outcomes related to self-management behavior across 8 dimensions. The heiQ has been found to capture dimensions strongly related to empowerment [14]. Of interest for our study, heiQ has been used in Denmark to evaluate the impact of telemedicine [15] and in Norway to access self-management in COPD [2].

The SF-36 was developed by the US Medical Outcomes Study to measure self-reported health status across 8 dimensions [16]. SF-36 is widely used and has been applied previously among patients with COPD and has identified psychological distress and poor health status when compared with the general population [17].

The aim of this study is three-fold. First, it is explored whether the clinical risk assessment of COPD is related to the individuals’ perception of living with their condition as measured with the following 3 PROMs: HLQ, heiQ, and SF-36. Thereafter, it is evaluated how the Epital living lab with its innovative reorganization of services influences participants’ perception of their condition measured by the 3 PROMs over a 12-month period. Finally, we explore whether clinical characteristics, commonly used by clinicians to evaluate the likelihood of deterioration or PROM scores capturing the participant’s perspective, predict the participants’ need of contact to the Epital response and coordination center.

## Methods

### Overview

This paper is part of a larger longitudinal study of the Epital living lab, which is described elsewhere [4]. In short, participants were recruited from the municipality of Lyngby-Taarbæk’s rehabilitation centers via leaflets at the local pharmacies and through the Danish Lung Association. In total, 93 individuals were included from April 2013 to December 2015; 61 women (mean age 74.3 years; range 47-91 years) and 32 men (mean age 73.0 years; range 49-87 years) participated. Data about exacerbations and number of contacts were first registered by November 2013. Hence, data about these 2 parameters are not available for the first 22 participants. Each participant was offered to be part of the Epital living lab for an unlimited period, and by the end of the study period, 66 participants were still enrolled. Participants were enrolled in average for 406 days (range 8-983 days).

Patients with a COPD diagnosis were included based on the criteria that they were able to cooperate and communicate using the Epital living lab equipment and had a Mini-Mental State Examination score above 22 [4]. The test is used to examine whether the cognitive function is sufficient to cooperate when using technology. Exclusion criteria were psychosis (including severe bipolar disease) or expected lifetime less than 90 days because of a diagnosed condition other than COPD, for example, cancer.

At the time of inclusion in the study, participants received a 2-hour visit from a medical doctor in their home where they were examined to verify the diagnosis of COPD. The severity of COPD was classified based on both spirometry to measure the airflow limitation (GOLD 1-4) and according to a risk assessment combining symptoms and exacerbation (GOLD A-B-C-D) and per the criteria of the GOLD Guidelines for COPD [3].

At entry to the study, the participants were examined by the Epital eDoctors and classified based on the number of exacerbations and hospital admissions during the last year before inclusion, symptoms, and the Medical Research Council breathlessness scale and lung function.

The initial spirometry, with measurements of the forced expiratory volume during the first second (FEV1), forced vital capacity, oxygen saturation, pulse, and temperature, was supervised by the eDoctor to serve as a baseline for the algorithm developed to be used by the participants to self-monitor their condition. Full datasets were only obtained for 88 of the 93 participants. The reason was that FEV1 is a mean value of first 3 measurements, which not all participants completed. A member of the municipality’s technical service team visited the participant and introduced the telemonitoring equipment: a tablet with applications for monitoring daily condition and communication with the response and coordination center by videoconference, a spirometer, a pulse oximeter, and a medical acute box [4].

Before the introduction to the equipment, the technical service team completed a mental score with the participant. The Mini Mental State Examination clarifies the mental state of the participants. If the score was above 22, the inclusion process was continued, and the participant filled in the PROMs (HeiQ, HLQ, and SF-36); if necessary, the participant was assisted by the technical service team. Filling in PROMs took approximately 30 to 50 min. If the participant appeared to be tired or not able to focus, the PROMs were collected at the next visit to avoid exhaustion. After 2 weeks, the participant was revisited by the technical service team to follow up on the usage of the equipment and to answer the participant’s questions. At baseline and at 6 and 12 months, the HLQ version 1.0DK, heiQ version 2.0DK, and the SF-36 version 1.1 DK were administered.

The HLQ consists of 9 dimensions, each ranging between 4 and 6 items [7]. The response options for dimensions 1 to 5 consist of a scale of values 1 to 4 (*strongly disagree* to *strongly agree*). Dimensions 6 to 9 consist of a difficulty scale from 1 to 5 (*cannot do* to *very easy*). Each dimension score was calculated as the mean of the items comprising the dimension (19).

The heiQ consists of 8 dimensions, with 4 to 6 items with response options ranging from 1 to 4 (*strongly disagree* to *strongly agree*). Each dimension score was calculated as the mean of the items comprising the dimension [6]. Dimension 3 (emotional distress) is normally reported as impact of distress, meaning a high score reflects a high impact of distress [6]. For this study, the scale was reversed for ease of interpretation. In this way, a high score means less distress and a low score more distress.

The SF-36 includes 8 multi-item scales, comprising a total of 36 items that assess health-related dimensions. The scoring further includes 2 components’ summary scores for physical and mental health [16]. To accommodate the multiple testing and reduce the risk of type 1 errors, we used only the 2 component summary scores for physical and mental health in the SF-36 questionnaire.

For all scales, missing items were imputed using the mean of other items in the dimension; however, if more than half of the items in a dimension were missing for an individual, the score for the dimension was regarded as missing.

A total of 431 questionnaires were completed, 237 (55%) under supervision and 178 (41%) without supervision (supervision status for 16 questionnaires was unregistered). Due to different inclusion periods, not all of the 93 participants completed questionnaires at 6 months and 12 months (Table 1). All questionnaires were completed between April 2013 and December 2015 except for 2 questionnaires, which were completed in January 2016. Due to a communication error with the technical service team, 15 questionnaires between January 2014 and May 2014 were not collected, resulting in a lower response rate at the 6-month follow-up.

**Table 1:** Questionnaire follow-up response rates.

Patient reported outcome measure	Baseline^a^, n (%)	6 months^a,b^, n (%)	12 months^a^, n (%)
Health Literacy Questionnaire	74 (81)	36 (40)	28 (31)
Health Education Impact Questionnaire	74 (81)	36 (40)	28 (31)
Short Form 36	85 (93)	42 (46)	28 (31)

^a^Response rate.

^b^Because of a communication error with the technical service team, 15 questionnaires covering the 6-month period were not collected between January 2014 and May 2014.

From the clinical program and database used in the Epital living lab (Epiprocess), data were available on sex, age, FEV1, exacerbations, and contacts with the Epital response and coordination center. Participants’ FEV1 was calculated as an average of the first 3 (supervised) spirometer tests. Percentage of expected FEV1 was based on participant’s age, sex, and height. The percentage was calculated with a predicted FEV1 based on data from a Danish norm population. Sufficient data were only available for 67 of the 88 patients grouped in the GOLD risk factor groups.

For the purpose of this study, we classified the participants as having an exacerbation when they were treated for a severe exacerbation through prescription of medication (addition of oral prednisolone, broad-spectrum antibiotics, and in addition short-acting beta-2-agonist). When a participant was in contact with the response and coordination center, the Epital nurse made a note; thus, the number of notes in the records was equal to the number of contacts with the response and coordination center.

Documentation of the participants’ exacerbations and contacts with the response and coordination center during the first 90 days of participation was available from November 2013 to December 2015. The participants’ exacerbations and contacts with the response and coordination center was documented for each participant for the period they participated. For analysis of relation between the GOLD risk factor and PROMs to contacts and exacerbation, only the data for the first 90 days were used; 90 days was chosen as a lower number of days were assumed to not detect a sufficient number of exacerbations, and a longer period may be influenced by the variations in the participants’ deterioration.

### Statistical Analysis

We reported outcome measure scores, FEV1, age, contacts, and exacerbations as means with SDs. The PROMs are reported as means and percentage of maximum score and range.

One-way analysis of variance (ANOVA) was used to test for differences in PROM score, FEV1, and age between risk groups. A posteriori test (Tukey honest significant difference method) was used to determine whether differences existed between severity groups.

Changes in scores of HLQ, heiQ, and SF-36 dimensions over the 6 and 12 months were modeled using linear mixed models to account for the correlation of repeated measurements within participants.

For the analysis of association between the number of contacts within the first 90 days of participation and FEV1, GOLD risk factor assessment, exacerbations, age, sex, and PROM scores, we calculated rate ratios with corresponding 95% CIs using quasi-Poisson regression models with a scale parameter to account for overdispersion.

For all tests, a significance level of .05 was used. To accommodate for multiple testing and reduce the risk of type 1 errors, we used only the 2-component summary scores for physical and mental health in the SF-36 questionnaire. The open source statistical program R version 0.98.1028 was used for the analysis.

### Ethics and Data Protection

The study was assessed and found not to need specific approval by the Regional office of the National Danish Ethics Committee (H-3-2012-FSP31). The program was also registered with the National Danish Data Agency by first the University of Copenhagen (2012-41-0384) and since January 2014 by the municipality of Lyngby-Taarbæk, Denmark (20150910229). All data were stored at the municipality and handled according to Danish legislation and regulations.

## Results

### Clinical Characteristics and Relationship to Health Literacy Questionnaire, Health Education Impact Questionnaire, and Short Form-36 Scores

The 93 participants recruited had a wide range of COPD severity (Table 2). There was no difference in age among the 4 risk assessment groups (A to D). The FEV1 differed across all risk assessment groups except for A, which did not differ from B and C (D and A, *P*<.001; C and B, *P*<.05; D and B, *P*<.001; D and C, *P*<.01).

In relation to the differences in PROM scores according to severity across the heiQ, HLQ, and SF-36 (19 dimensions), only 3 dimensions showed significant differences between groups (Table 2): heiQ3 (emotional distress) (D and C, *P*<.05), heiQ5 (constructive attitudes and approaches; D and C, *P*<.01) and SF-36 Physical health (D and A, *P*<.001; D and B, *P*<.01), indicating more distress, poorer attitudes, and poorer health status with increasing severity. It should be noted that heiQ3 (emotional distress) in group C was higher than the other groups, indicating less distress for this group. A similar but not significant pattern was seen in SF-36 Mental health, supporting this reduced burden of the condition in group C. FEV1, which is a direct measure of pulmonary function, declined with increasing GOLD risk assessment score.

**Table 2:** Baseline mean and standard deviation across chronic obstructive pulmonary disease severity.

Clinical characteristics and patient-reported outcome measure dimensions	Risk factor					Difference
	A (N=11)	B (N=23)	C (N=13)	D (N=41)	Total (N=88)	
Age (years), mean (SD)	73.1 (6.3)	72.2 (10.1)	74.8 (13.3)	74.7 (10.3)	73.9 (10.1)	—^a^
FEV1^b^ (L), mean (SD)	1.47 (0.46)	1.52 (0.45)	1.15 (0.21)	0.75 (0.27)	1.11 (0.5)^c^	(A;B); (B;C); (B;D); (C;D)^d^
Expected FEV1^e^, n (%)	61 (10)	61(7)	44(8)	35 (17)	45 (18)	—
HLQ1^f^: Feeling understood and supported by health care providers, mean (SD)	3.05 (0.23)	2.95 (0.63)	3.04 (0.46)	3.06 (0.54)	3.04 (0.51)	—
HLQ2: Having sufficient information to manage my health, mean (SD)	2.93 (0.37)	2.90 (0.53)	2.85 (0.36)	3.00 (0.51)	2.95 (0.47)	—
HLQ3: Actively managing my health, mean (SD)	2.73 (0.44)	2.72 (0.54)	2.78 (0.34)	2.96 (0.34)	2.84 (0.42)	—
HLQ4: Social support for health, mean (SD)	2.89 (0.59)	2.91 (0.58)	3.18 (0.42)	2.93 (0.56)	2.96 (0.54)	—
HLQ5: Appraisal of health information, mean (SD)	2.75 (0.44)	2.73 (0.63)	2.60 (0.55)	2.66 (0.47)	2.70 (0.53)	—
HLQ6: Ability to actively engage with health care providers, mean (SD)	3.98 (0.28)	3.84 (0.53)	3.75 (0.40)	3.83 (0.51)	3.85 (0.46)	—
HLQ7: Navigating the health care system, mean (SD)	3.73 (0.56)	3.59 (0.50)	3.47 (0.35)	3.66 (0.56)	3.62 (0.50)	—
HLQ8: Ability to find good health information, mean (SD)	3.87 (0.49)	3.82 (0.59)	3.58 (0.37)	3.74 (0.40)	3.75 (0.46)	—
HLQ9: Understanding health information well enough to know what to do, mean (SD)	3.82 (0.36)	3.85 (0.52)	3.80 (0.27)	3.91 (0.41)	3.87 (0.41)	—
heiQ1^g^: Health directed activities, mean (SD)	3.09 (0.56)	2.85 (0.69)	3.15 (0.74)	2.73 (0.77)	2.89 (0.72)	—
heiQ2: Positive and active engagement in life, mean (SD)	2.98 (0.55)	3.18 (0.57)	3.13 (0.42)	2.99 (0.43)	3.07 (0.48)	—
heiQ3: Emotional distress, mean (SD)	2.74 (0.63)	2.67 (0.72)	3.07 (0.68)	2.34 (0.68)	2.61 (0.71)^h^	(C;D)^i^
heiQ4: Self-monitoring and insight, mean (SD)	3.03 (0.32)	3.02 (0.42)	3.14 (0.49)	3.12 (0.41)	3.08 (0.40)	—
heiQ5: Constructive attitudes and approaches, mean (SD)	3.13 (0.46)	3.15 (0.50)	3.33 (0.38)	2.78 (0.50)	3.02 (0.51)^j^	(C;D)^k^
heiQ6: Skill and technique acquisition, mean (SD)	3.11 (0.39)	2.93 (0.43)	3.00 (0.52)	2.95 (0.41)	2.98 (0.42)	—
heiQ7: Social integration and support, mean (SD)	3.05 (0.43)	3.03 (0.56)	3.07 (0.52)	3.03 (0.54)	3.04 (0.51)	—
heiQ8: Health services navigation, mean (SD)	3.24 (0.39)	3.28 (0.41)	3.40 (0.44)	3.23 (0.54)	3.28 (0.47)	—
SF-36: Physical, mean (SD)	45.9 (8.1)	40.5 (10.8)	39.3 (9.7)	31.7 (7.5)	37.2 (10.1)^b^	(A;D) (B;D)^l^
SF 36: Mental, mean (SD)	49.5 (14.9)	46.5 (16.0)	53.3 (14.1)	40.8 (13.3)	45.8 (10.1)	—

^a^No difference.

^b^FEV1: forced expiratory volume during the first second.

^c^*P*<.001.

^d^Regarding FEV1, all GOLD risk factor groups differ from each other except for A, which did not differ from B and C. D and A, *P*<.001; C and B, *P*<.05; D and B, *P*<.001; D and C, *P*<.01.

^e^Number in GOLD risk factor groups: A=9, B=15, C=9, and D=34.

^f^HLQ: Health Literacy Questionnaire.

^g^heiQ: Health Education Impact Questionnaire.

^h^*P*<.05.

^i^The difference is between C and D, *P*<.05.

^j^*P*<.01.

^k^The difference is between C and D, *P*<.01

^l^The difference is between A and D, *P*<.001; D and B, *P*<.01

Using correlation test, FEV1 was found to be positively associated with heiQ2 (positive and active engagement in life, *P*<.05), heiQ3 (emotional distress, *P*<.05), heiQ5 (constructive attitudes and approaches, *P*<.001), and heiQ8 (health service navigation, *P*<.05) but also with SF-36 Physical health (*P*<.001). Furthermore, we found a negative association between FEV1 and HLQ3 (actively managing my health, *P*<.05).

Over a 12-month period, various changes in PROMs were found. After 6 months, an increase was observed for heiQ6 (skill and technique acquisition) as well as in the reversed heiQ3 (emotional distress). The latter effect persisted after 12 months. HeiQ4 (self-monitoring and insight) also increased slightly. Meanwhile, HLQ3 (actively managing my health) decreased after 6 and 12 months. No changes in the SF-36 mental and physical components were observed (Table 3).

**Table 3:** Change in patient-reported outcome measures over 6 and 12 months using linear mixed models to account for the correlation of repeated measurements within participants.

Patient-reported outcome measures	Baseline (N=74)^a^, mean	6 months (N=36)^b^		12 months (N=28)	
		Mean	Mean baseline to 6-month difference (95% CI)	Mean	Mean baseline to 12-month difference (95% CI)
HLQ1^c^: Feeling understood and supported by health care providers^d^	3.0	3.0	−0.04 (−0.22 to 0.13)	3.0	0.00 (−0.19 to 0.19)
HLQ2: Having sufficient information to manage my health^d^	2.9	3.0	0.03 (−0.12 to 0.17)	2.9	0.00 (−0.15 to 0.16)
HLQ3: Actively managing my health^d^	2.8	2.8	−0.06 (−0.16 to 0.05)	2.7	−0.14^e^ (−0.25 to −0.02)
HLQ4: Social support for health^d^	3.0	3.0	0.04 (−0.11 to 0.18)	2.9	−0.06 (−0.22 to 0.09)
HLQ5: Appraisal of health information^d^	2.7	2.8	0.06 (−0.08 to 0.21)	2.6	−0.08 (−0.23 to 0.08)
HLQ6: Ability to actively engage with health care^f^ providers	3.8	3.9	0.10 (−0.07 to 0.27)	3.7	−0.07 (−0.26 to 0.12)
HLQ7: Navigating the health care system^f^	3.6	3.6	0.01 (−0.17 to 0.18)	3.6	−0.04 (−0.24 to 0.14)
HLQ8: Ability to find good health information^f^	3.7	3.7	−0.04 (−0.23 to 0.13)	3.6	−0.10 (−0.29 to 0.10)
HLQ9: Understanding health information well enough to know what to do^f^	3.9	3.9	0.00 (−0.16 to 0.16)	3.9	−0.05 (−0.22 to 0.13)
heiQ1^g^: Health directed activities^d^	2.9	2.9	−0.01 (−0.21 to 0.19)	2.8	0.06 (−0.17 to 0.28)
heiQ2: Positive and active engagement in life^d^	3.1	3.1	−0.03 (−0.17 to 0.10)	3.1	0.05 (−0.10 to 0.20)
heiQ3: Emotional distress^d,h^	2.6	2.8	0.24^i^ (0.08 to 0.40)	2.8	0.20^e^ (0.02 to 0.38)
heiQ4: Self-monitoring and insight^d^	3.0	3.0	0.01 (−0.14 to 0.15)	3.2	0.16^e^ (0.00 to 0.32)
heiQ5: Constructive attitudes and approaches^d^	3.0	3.1	0.08 (−0.07 to 0.23)	3.1	0.06 (−0.10 to 0.23)
heiQ6: Skill and technique acquisition^d^	2.9	3.1	0.17^e^ (0.02 to 0.33)	3.0	0.14 (−0.04 to 0.32)
heiQ7: Social integration and support^d^	3.0	3.0	−0.05 (−0.18 to 0.07)	3.0	−0.03 (−0.17 to 0.11)
heiQ8: Health services navigation^d^	3.2	3.3	0.05 (−0.10 to 0.21)	3.2	0.02 (−0.15 to 0.20)
SF-36^j^: Physical	37.2	37.2	−0.01 (−2.5 to 2.5)	36.1	−1.1 (−4.0 to 1.8)
SF-36: Mental	45.5	47.3	1.8 (−1.8 to 5.4)	47.0	1.5 (−2.6 to 5.7)

^a^N=82 for SF-36 Physical SF-36 Mental.

^b^N=42 for SF-36 Physical SF-36 Mental.

^c^HLQ: Health Literacy Questionnaire.

^d^Range 1-4.

^e^*P*<.05.

^f^Range 1-5.

^g^heiQ: Health Education Impact Questionnaire.

^h^Scale reversed, that is, high score is low emotional distress.

^i^*P*<.01.

^j^SF-36: Short-Form-36.

### Association Between Patient-Reported Outcome Measures and the Number of Contacts to the Response and Coordination Center

As seen in Table 4, the number of contacts and exacerbations increased with increasing COPD severity. There was a positive association between severity and contacts (*P*<.05) and severity and exacerbations (*P*<.001), respectively.

To determine whether the number of contacts is associated with clinical characteristics or PROM scores at the baseline, a quasi-Poisson regression model was constructed (Table 5).

Of the clinical characteristics, the number of exacerbations and severity expressed as the GOLD risk factor assessment predicted the number of contacts during the 90 days.

There was no association between baseline-FEV1, sex, or age in relation to the number of contacts.

Of the PROMs, we found that an increase in HLQ3 (actively managing my health) predicted higher number of contacts (*P*<.05); however, adjustment for severity proved this effect insignificant. In contrast, SF-36 Physical health component score predicted a decrease in the number of contacts, but it was also found to be insignificant after adjustment for severity of COPD. Higher baseline scores on heiQ6 (skill and technique acquisition) and HLQ8 (ability to find good health information) predicted lower number of contacts. The association between number of contacts and heiQ6 was significant both with and without adjustment for severity, whereas the association with HLQ8 was only significant after adjustment.

There were no associations between PROM scores at entry and exacerbations over the 90 days (data not shown).

**Table 4:** Number of contacts with the response and coordination center and registered severe exacerbations grouped by chronic obstructive pulmonary disease severity over 90 days.

Number	A	B	C	D	Total
Contacts, mean (SD)	6.0 (2.8)	7.6 (6.6)	12.0 (20.1)	15.9 (16.0)	11.6 (13.8)
Exacerbations, mean (SD)	0.0 (0.0)	0.21 (0.42)	0.42 (1.13)	0.54 (0.83)	0.36 (0.72)

**Table 5:** Association between clinical characteristics, patient-reported outcome measures, and number of contacts during the first 90 days after entry (rate ratios and 95% CIs). Column 2 presents the unadjusted rate ratio and column 3 gives rate ratio adjusted for GOLD severity. Rate ratio greater than 1 indicates a higher frequency and lesser than 1 a lower frequency in number of contacts with increased values of the variable.

Variable	Baseline prediction of number of future contacts over 90 days, rate ratio (95% CI)	
	Unadjusted	Adjusted for GOLD^a^ severity score
FEV1^b^	0.76 (0.42-1.32)	1.96 (0.92-4.02)
GOLD severity score	0.71^c^ (0.54-0.90)	NA^d^ [variable used as covariate]
Sex (1=men, 0=female)	1.04 (0.56-1.85)	1.13 (0.64-1.93)
Age (years)	0.99 (0.96-1.02)	0.99 (0.96-1.01)
Exacerbations (number)	1.99^e^ (1.64-2.40)	1.89^e^ (1.54-2.31)
HLQ1^f^: Feeling understood and supported by health care providers	1.14 (0.63-2.14)	0.97 (0.57-1.70)
HLQ2: Having sufficient information to manage my health	1.18 (0.63-2.21)	0.93 (0.52-1.68)
HLQ3: Actively managing my health	1.94^c^ (1.02-3.71)	1.47 (0.75-2.86)
HLQ4: Social support for health	1.02 (0.58-1.76)	0.92 (0.54-1.55)
HLQ5: Appraisal of health information	1.24 (0.70-2.17)	1.17 (0.70-1.94)
HLQ6: Ability to actively engage with health care providers	0.85 (0.46-1.61)	0.87 (0.50-1.52)
HLQ7: Navigating the health care system	0.88 (0.52-1.53)	0.81 (0.51-1.34)
HLQ8: Ability to find good health information	0.55 (0.30-1.00)	0.55^c^ (0.32-0.96)
HLQ9: Understanding health information well enough to know what to do	0.80 (0.41-1.57)	0.72 (0.39-1.33)
heiQ1^g^: Health directed activities	0.72 (0.47-1.08)	0.78 (0.54-1.13)
heiQ2: Positive and active engagement in life	0.92 (0.52-1.62)	0.94 (0.54-1.62)
heiQ3: Emotional distress^h^	0.81 (0.56-1.18)	0.90 (0.63-1.28)
heiQ4: Self-monitoring and insight	1.35 (0.68-2.60)	1.18 (0.62-2.20)
heiQ5: Constructive attitudes and approaches	0.62 (0.36-1.07)	0.74 (0.43-1.26)
heiQ6: Skill and technique acquisition	0.47^c^ (0.26-0.86)	0.49^c^ (0.29-0.84)
heiQ7: Social integration and support	0.76 (0.43-1.34)	0.73 (0.43-1.23)
heiQ8: Health services navigation	0.64 (0.34-1.18)	0.67 (0.40-1.14)
SF-36^i^: Physical	0.96^j^ (0.94-0.99)	0.98 (0.95-1.00)
SF 36: Mental	0.98 (0.96-1.01)	0.99 (0.97-1.01)

^a^GOLD: Global Initiative for Chronic Obstructive Lung Disease.

^b^FEV1: forced expiratory volume during the first second.

^c^*P*<.05.

^d^N/A: not applicable.

^e^*P*<.001.

^f^HLQ: Health Literacy Questionnaire.

^g^heiQ: Health Education Impact Questionnaire.

^h^Scale reversed, that is, high score is low emotional distress.

^i^SF-36. Short Form-36.

^j^*P*<.01.

## Discussion

In this study, we have systematically explored, prospectively, the utility of 3 PROMs to evaluate the impact of living with COPD and how an innovative reorganization of services improves participants’ understanding, self-management, and well-being. We used 3 psychometrically robust PROMs that have previously been used to assess health literacy, empowerment, and self-management in COPD and self-reported physical and mental health.

When this study was initiated in 2013, there was limited literature on the HLQ and heiQ in relation to chronic conditions, such as COPD. Given that the Epital living lab was a substantial clinical and self-care change for patients, we regarded it as critical to explore the participants’ severity of COPD in relation to their reported impact of living with the condition and how this changed over time; hence, we employed the HLQ, heiQ, and SF-36 to assist with this task.

### Characteristics of Participants in Relation to Severity of COPD

The increase in skills and behavior related to being more active in self-managing while experiencing a highly active disease is likely to reflect direct learning over time from closer contact and regular interactions with the health care services and through increasing competence in engaging with the technology. Our data show that those who had increased contact with the 24/7 response and coordination center tended to report a decrease in heiQ3 (emotional distress) as well as a higher heiQ6 (skill and technique acquisition) at entry to the study.

Overall, there was a tendency for those in risk assessment group C, the second highest category, to report a lower burden related to COPD compared with the other groups. This was evident through heiQ3 (emotional distress) and SF-36 Mental health scores. This may be explained by the fact that COPD patients in group C, and also D, were followed up by hospital specialists in Denmark, in contrast to patients in groups A and B, who were followed up by their general practitioners. The poorer mental well-being of group D may reflect the very poor and debilitating health status they experience that is not alleviated by specialist contacts. This is only speculative and needs to be confirmed in further studies, examining a larger sample and also applying a mixed-methods approach. Recently, a Norwegian study examined the relation between heiQ domains, burden of the condition, and severity in people with COPD. In agreement with our findings, they demonstrated high distress but in contrast to our study, also showed that all 8 heiQ dimensions, except for the heiQ4 (self-monitoring and insight), were directly associated with the burden of living with COPD [2].

### The Impact of the Intervention Over a 6- and 12-Month Period

The 24/7 access to health resources provided by the Epital living lab was associated with a reduction in the burden of the condition (lower emotional distress—ie, reduced negative affective responses to the disease). This may be due to their knowledge that assistance and treatment options are always only one click away. The training during and after the inclusion and the regular use of the technology may also have resulted in an increase in both heiQ4 (self-monitoring and insight) and heiQ6 (skill and technique acquisition) as the participants acquire confidence in using the technical solution with the tablet, apps, pulse oximeter, and spirometer. Surprisingly, HLQ3 (actively managing my health) declined over the period. This may be related to the intervention being on hand 24/7. It is possible that some reduced vigilance in self-care may have occurred given the constant presence of strong and immediate support and security.

The finding that heiQ3 (emotional distress) and heiQ4 (self-monitoring and insight) increase over 12 months are in line with a regional study in Denmark with telemedicine interventions regarding COPD where 3 heiQ dimensions were found to increase over a 6-month period: heiQ3 (emotional distress), heiQ4 (self-monitoring and insight), and heiQ8 (health services navigation) [15], with the first 2 being in accordance with our results. Interestingly, we also found an increase in heiQ6 (skill and technique acquisition) but not in heiQ8 (health services navigation). This may be explained by differences in the design between the studies. In the Epital living lab, participants had continuous access to the services and were supported by municipality nurses, who were in turn, supported by eDoctors. The other study did not offer daily access and was administered by a hospital. These findings indicate that digitally supported health services may decrease the distress and relieve the burden of COPD and contribute to better self-management, self-monitoring, and insight.

### Predictors of Number of Contacts During the First 90 Days

As anticipated, the severity of the condition as estimated by the GOLD risk assessment groups predicted the number of contacts with the response and coordination center during the first 90 days.

This may be the result of the high number of exacerbations found in the higher risk groups. This supports the GOLD risk factor assessment as a useful tool to predict necessary resources [3]. Interestingly, the participants’ FEV1 at entry did not correlate to the number of contacts. This supports the intent of the new GOLD risk assessment, as FEV1 is not an essential indicator for the need of contacts.

Only 2 of the PROMs, heiQ6 (skill and technique acquisition) and HLQ8 (ability to find good health information), were independently associated with the number of contacts after a correction for severity of the condition. Higher scores for these 2 domains were related to a lower number of contacts, which suggests that development of skills to handle the condition and ability to find information reduces the need for assistance from the response and coordination center. It may be of value to focus on these competences for people living with COPD to reduce the burden of disease they experience.

Interestingly, our findings suggest that selected PROMs can contribute to a richer understanding of how participants are influenced by their condition. An example is how the HLQ3 (actively managing my health) is higher in GOLD group D, indicating that a higher number of contacts to the health professionals and over time decreases when participating in the Epital living lab intervention. This indicates that this PROM may capture a new perspective on the burden of the condition.

Overall, our results demonstrate that selected dimensions from heiQ and HLQ may be used to better understand people living with COPD and how a digitally assisted intervention affects them with less emotional distress, acquired skills to handle the condition, and at the same time, an indication of feeling less actively involved. Whether the latter is due to a supportive environment or relief of the burden of the condition remains to be further investigated in larger studies supplemented with inclusion of interviews and observations. In pursuing the idea of using selected dimensions from heiQ and HLQ, we developed a new instrument inspired by our findings from this study and other reports, where we combine heiQ dimensions 3 to 6 and HLQ dimensions 1 and 4 with the 7 dimensions of the recently developed eHealth Literacy Questionnaire (eHLQ), resulting in an instrument to measure readiness and enablement of technology called Readiness and Enablement Index for Health Technology [18].

### Limitations of the Study

The investigators expected that the participants in the living lab would develop their knowledge, skills, attitudes, and motivation during the study. The relatively few dimensions, which increased and the decreased in HLQ3 (actively managing my health), was unexpected. It is likely that we in the implementation of the Epital living lab as an action research project focused too much on the technical setup, stability and usability of solutions, and the medical support when needed. We therefore recommend that when the Epital living lab is implemented at other sites, more attention should be paid to the development of activities strengthening the participants’ understanding of how to live with COPD and other conditions. Examples are educational and training programs, coaching, and other tools such as the guided self-determination program [19,20], which is designed to increase life skills and empower the participants.

Another limitation of the study was that the number of participants was small, and it was not possible to design a control study or match with controls in other studies. There is a risk of type 2 error because of the limited number of participants and the further reduction of participants after 6 and 12 months.

The convenience sampling in a community with a relatively high average income is also a weakness, limiting generalization to other settings. However, given the aims of the study, we expect the data are internally valid and provide reasonable guidance regarding selection of PROMs for wider study of the Epital living lab.

A final weakness is that we were not able to include some of the most recent PROMs that measure eHealth literacy at the time of the data collection. A recently published tool, the eHLQ, may have provided clearer indicators of participants’ ability to engage with the technology and reasons for limited participation and consequent well-being improvement [21].

### Conclusions

In this study, we demonstrated how the HLQ, heiQ, and SF-36 can be used as PROMs in relation to COPD to provide researchers and clinicians with greater insight into how this condition affects individuals’ ability to understand and manage their condition and how they perceive their physical and mental health. The PROMs add to the information obtained with the clinical characteristics including the GOLD risk assessment.

At baseline, the PROM data from the GOLD risk group D provided a more nuanced picture of how living with COPD affects their well-being. The combination of self-reported increased emotional distress, lower self-reported physical health, and a less constructive approach and attitude to their condition provides a clear impression of how frail they are.

The PROMs also help to understand how the Epital living lab affects the participants over a 12-month period, including the potential for increasing skills and at the same time reducing both emotional distress and the perception of being active in managing their condition.

## Abbreviations

ANOVA: analysis of variance
COPD: chronic obstructive pulmonary disease
eHLQ: eHealth Literacy Questionnaire
FEV1: forced expiratory volume during the first second
GOLD: Global Initiative for Chronic Obstructive Lung Disease
heiQ: Health Education Impact Questionnaire
HLQ: Health Literacy Questionnaire
PROM: patient-reported outcome measure
SF-36: Short Form-36
